# Physical Fitness Benchmarks for Preschool Children in South Korea: A Population-Based Study

**DOI:** 10.3390/children12030361

**Published:** 2025-03-14

**Authors:** Moon-Yeon Oh, Jae-Ho Lee

**Affiliations:** 1 Department of Pediatrics, Seoul St. Mary’s Hospital, College of Medicine, The Catholic University of Korea, Seoul 06591, Republic of Korea; moonicu@catholic.ac.kr; 2 Graduate School, University of Ulsan College of Medicine, Ulsan 44033, Republic of Korea; 3 Department of Emergency Medicine, Asan Medical Center, University of Ulsan College of Medicine, Seoul 05505, Republic of Korea

**Keywords:** physical fitness, preschool children, reference standard

## Abstract

**Background**: Physical fitness is vital for children’s development and future health. However, Asian preschool-aged populations lack robust physical fitness benchmarks. **Objectives**: This study aimed to establish age- and sex-specific physical fitness reference values for Korean preschool children and examine associations with anthropometric measures. **Methods**: A retrospective analysis of data from 36,118 children aged 3 to 6 years was conducted, using five physical fitness tests (Sit and Reach Test, V-Sit Endurance Test, Standing Long Jump, Single-Leg Stand, and 5-Meter Shuttle Run). Percentile curves were generated via Generalized Additive Models for Location, Scale, and Shape (GAMLSS). Relationships between fitness and anthropometric measures were analyzed with LOESS regression. **Results**: Girls outperformed boys in flexibility, endurance, and balance, while boys excelled in jumping power. Physical fitness improved with age, with performance peaking near average height and weight z-scores. **Conclusions**: This study provides the first large-scale reference standards for physical fitness in Korean preschoolers, facilitating early identification of fitness deficits and guiding interventions.

## 1. Introduction

Physical fitness is a crucial indicator of children’s current and future health. Numerous studies have linked physical fitness to preventing chronic conditions, such as obesity, cardiovascular disease, and mental health issues, emphasizing its role in promoting long-term health and well-being [1,2,3]. In children, higher levels of physical fitness are associated with lower adiposity, healthier cardiovascular profiles, and enhanced motor skills, highlighting its importance from an early age [1,2,4,5]. In particular, preschool years represent a critical window for motor development and establishing lifelong physical activity habits. Therefore, efforts are being made to assess children’s physical fitness early to positively impact their long-term health and development [6,7]. The preschool years are a period of rapid growth and development, making it essential to establish appropriate age-specific reference standards to accurately assess children’s physical fitness levels.

While reference values for physical fitness have been established for older children in various countries, preschool-age children have not been as comprehensively studied. European studies, such as the IDEFICS and PREFIT projects, have provided significant insights, offering sex- and age-specific fitness standards for European preschool children [8,9]. Although several studies on preschool-aged children’s physical fitness exist in Asia, and some research in China has reported mean values by age, comprehensive percentile-based standard reference values remain limited [10,11,12]. Furthermore, due to cultural and developmental variations, region-specific benchmarks are necessary, as existing European standards may not be directly applicable to Asian populations.

This study aimed to provide age- and sex-specific physical fitness reference values for Korean preschool children. Additionally, it examined differences in physical fitness by age and sex, and its relationship with basic anthropometric measures, including height, weight, and body mass index (BMI), to better understand physical development in this age group. By providing comprehensive benchmarks, this study contributes to early intervention strategies, enabling educators and healthcare professionals to monitor physical development and implement targeted health policies for young children.

## 2. Methods

### 2.1. Study Design

This retrospective analysis used anonymized anthropometric data and physical fitness test results from the Seoul Metropolitan Government and local public health centers. Since 2013, Seoul has implemented a public health initiative to assess the growth and physical fitness of children enrolled in daycare centers once or twice a year. Assessments include anthropometric measurements (age in months, height, weight, and BMI) and five physical fitness tests: the Sit and Reach Test (SRT), V-Sit Endurance Test (VSE), Standing Long Jump (SLJ), Single-Leg Stand (SLS), and 5-Meter Shuttle Run (5SR). Due to the retrospective design of this study, the available data were limited to participants’ age in months, sex, anthropometric measurements, and physical fitness results provided by the Seoul Metropolitan Government.

This study analyzed data from 39,510 children, with 29,362 measurements taken between March 2017 and December 2019 and 10,148 measurements taken between October 2022 and January 2023. The program was suspended from 2020 to September 2022 due to the COVID-19 outbreak.

The study was approved by the Ethics Committee of Seoul St. Mary’s Hospital, The Catholic University of Seoul, Korea (KC24ZISI0178). Due to the study’s retrospective nature, the Institutional Review Board of Seoul St. Mary’s Hospital waived the need for informed consent.

### 2.2. Study Population

Out of the total 39,510 test results, we excluded 3232 entries with missing information on age in months or sex and 160 entries for children younger than 36 months or older than 84 months. This study analyzed the remaining 36,118 children’s data, collected through a large-scale government program, representing one of the most comprehensive datasets for preschool fitness in Asia.

Erroneous measurements were removed, and Z-scores by age in months were calculated for each test result. Data with Z-scores exceeding ±3 were considered outliers and excluded from the analysis.

**Figure 1:**
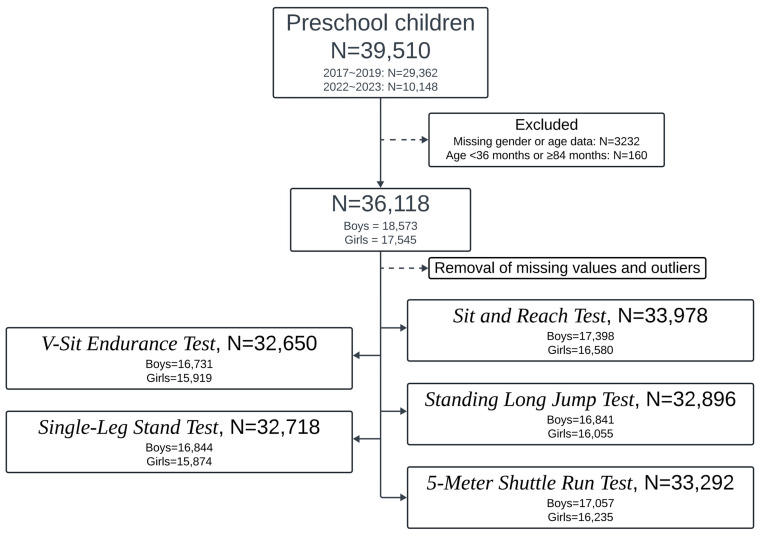
Flow chart of the population involved in this study.

As not all children completed every test, the final sample size varied across test items, ranging from 30,511 to 33,978. The study population flow and sample sizes for each test item are illustrated in Figure 1.

### 2.3. Anthropometric Measurements

Height and weight were measured using a stadiometer and weighing scale, respectively, and recorded in centimeters (cm) and kilograms (kg) to the nearest 0.1 cm and 0.1 kg. BMI was calculated by dividing the weight (kg) by the square of the height (m^2^). To analyze the relationship between anthropometric measurements and physical fitness, anthropometric variables (height, weight, and BMI) were standardized using age-specific Z-scores [13].

### 2.4. Physical Fitness Measurements and Significance

Five tests were conducted to assess children’s physical fitness: SRT, VSE, SLJ, SLS, and 5SR. These tests measured flexibility, abdominal endurance, jumping power, balance, and agility. These abilities are directly related to children’s development and growth and hold significant importance for several reasons. The tests were selected based on their validity in assessing core fitness components in young children [12,14], and this test battery is commonly used for physical fitness assessment in Seoul, South Korea [15]. A summary of these tests is provided in Table 1.

(1) Sit and Reach Test (SRT)

The SRT used a flexibility measurement device. Children sat with legs fully extended and feet flat against the apparatus. With knees straight, they placed their hands together, extended their arms forward at chest height, and slowly leaned forward, sliding their hands along the measurement board. The distance from the fingertips to the soles of the feet was measured to the nearest 0.1 cm at the point where they held the maximum position for 2 s. Participants were instructed to avoid bending their knees, bouncing, or using one hand to push.

The SRT evaluates flexibility, particularly in the hamstrings and lower back. Assessing these areas provides insights into injury prevention and the potential to enhance the range of motion. Moreover, appropriate flexibility supports the maintenance of a healthy posture and encourages active participation in physical activities [16,17].

(2) V-Sit Endurance Test (VSE)

For the VSE, participants sat on a mat with legs straight and feet together, raising their arms to shoulder height in a forward position. Upon hearing the start signal, they leaned back slightly while lifting their feet off the ground, maintaining a “V” position. The time they could sustain this position was recorded to the nearest 0.1 s. Participants practiced holding the posture, ensuring their feet stayed together and their knees remained straight. The measurement ended when the feet touched the ground or dropped within 10 cm of it. The test was conducted once.

The VSE assesses abdominal endurance and the strength of the spinal erector muscles. This test measures the endurance of the abdominal and lower back muscles, which are essential for stability and posture maintenance. These metrics are key indicators of a child’s physical stability and overall health [17,18].

(3) Standing Long Jump (SLJ)

The SLJ required participants to align their toes at a baseline and jump forward with both feet together, aiming for maximum distance. The distance from the baseline to the rearmost heel touching the ground was measured to the nearest 0.1 cm. Participants practiced the jump, and the test was conducted twice, with the better score recorded.

The SLJ evaluates explosive lower-body strength, measuring the power and coordination of the leg muscles. It assesses foundational physical abilities and provides critical information on muscle strength improvement and motor skill development during growth. Lower-body strength is crucial for activities such as running, jumping, and other forms of physical movement, contributing to overall physical competence and self-confidence in children [17,19,20,21].

(4) Single-Leg Stand (SLS)

In the SLS, participants extended their arms sideways and, upon hearing the start signal, lifted one leg, bending it at a 90-degree angle, and balanced on the other leg. The duration they maintained the position was recorded to the nearest 0.1 s. Participants were instructed to avoid touching the bent leg to their body. The test ended when the raised leg touched the ground, or the supporting leg moved from its position. The test was conducted once.

The SLS measures balance by assessing the capacity to maintain balance while standing on one leg. Balance is fundamental for posture control and sustaining motor functions, reflecting the precision and stability of body movements. Balance strengthens the growing nervous system and coordination, improving motor control and physical stability in daily activities [17,22,23].

(5) Five-Meter Shuttle Run (5SR)

The 5SR involved participants running back and forth between a starting line and a point 5 m away, where two wooden blocks were placed. They retrieved the blocks one by one and put them in the designated area at the starting line. The total task time was recorded to the nearest 0.1 s. The test was conducted once.

The 5SR evaluates agility and cardiorespiratory endurance. It measures physical agility and cardiovascular function by assessing the ability to change direction quickly over short distances. These metrics are essential for determining children’s physical efficiency and fitness levels. Agility and cardiorespiratory endurance enable children to participate in various physical activities, promote weight management, enhance cardiovascular health, and positively influence continuous growth and development [1,17,24,25,26].

### 2.5. Statistical Analysis

Physical fitness measurements were analyzed by calculating the mean, standard deviation, and 95% confidence intervals for each fitness variable across age and sex groups. To generate percentile curves for physical fitness variables across age and sex, we used the Generalized Additive Model for Location, Scale, and Shape (GAMLSS) method. The analysis used the GAMLSS package (version 5.4-22) in R (version 4.4.1). GAMLSS allows the modeling of up to four distribution parameters: Location (μ), Scale (σ), skewness (ν), and kurtosis (τ), enabling flexible modeling of complex distributions. This method extends the LMS approach and provides a more robust fit by considering the influence of age on the distribution parameters. GAMLSS is particularly suited for modeling non-linear age trends and non-normally distributed physical fitness variables, ensuring accurate representation of developmental patterns.

Various distributions, including Box–Cox Cole and Green (BCCG), Box–Cox t (BCT), Box–Cox Power Exponential (BCPE), and Generalized Gamma (GA), were fitted to the data to model each physical fitness outcome. The choice of distributions was based on their suitability for each variable and the nature of the data.

The effect of age on the parameters was modeled using penalized B-splines to capture non-linear age effects, allowing for smooth transitions between age groups. This approach provided flexibility in fitting the curves to the observed data patterns.

Goodness of fit was evaluated using the Bayesian Information Criterion (BIC) and diagnostic tools like worm plots and Q-Q plots. The BIC was used to compare the models, and the model with the lowest BIC was selected as the final model. Worm plots were employed as a diagnostic tool to verify the adequacy of kurtosis adjustment and assess the fit of the chosen distribution. The Q-Q plots provided an additional check for the appropriateness of the fitted models.

Percentile curves for the 1st, 3rd, 10th, 25th, 50th, 75th, 90th, 97th, and 99th percentiles were derived based on the best-fitting models. These curves allow the classification of children’s physical fitness across different age groups and sexes. Percentiles were calculated for key age points (36, 48, 60, 72, and 84 months), providing reference standards for growth and fitness trends in preschool children. The results offer detailed insights into the distribution of fitness parameters and enable comparisons between different age groups and sexes.

Outliers were managed using robust distributions such as BCCG and BCPE, which are designed to handle deviations effectively. Missing values were excluded from the analysis to ensure the accuracy of the derived percentile curves.

This approach draws on similar studies that used GAMLSS to generate reference standards for children’s physical health [8,9].

Each fitness metric was normalized and expressed as an age-specific z-score to account for developmental differences across ages, facilitating standardized comparison across metrics. Anthropometric indicators, including height, weight, and BMI, were similarly transformed into age-specific z-scores to control for age-related growth and development, enabling accurate assessment of relative standing within age cohorts and an in-depth analysis of the relationships between anthropometric measurements and physical fitness.

The relationships between each fitness metric and anthropometric indicator were analyzed using locally weighted scatterplot smoothing (LOESS) regression models. This non-parametric approach was selected to model potential non-linear relationships while minimizing assumptions about data distribution. LOESS provides a flexible framework for modeling the association between anthropometric measures and physical fitness.

## 3. Results

**Figure 2:**
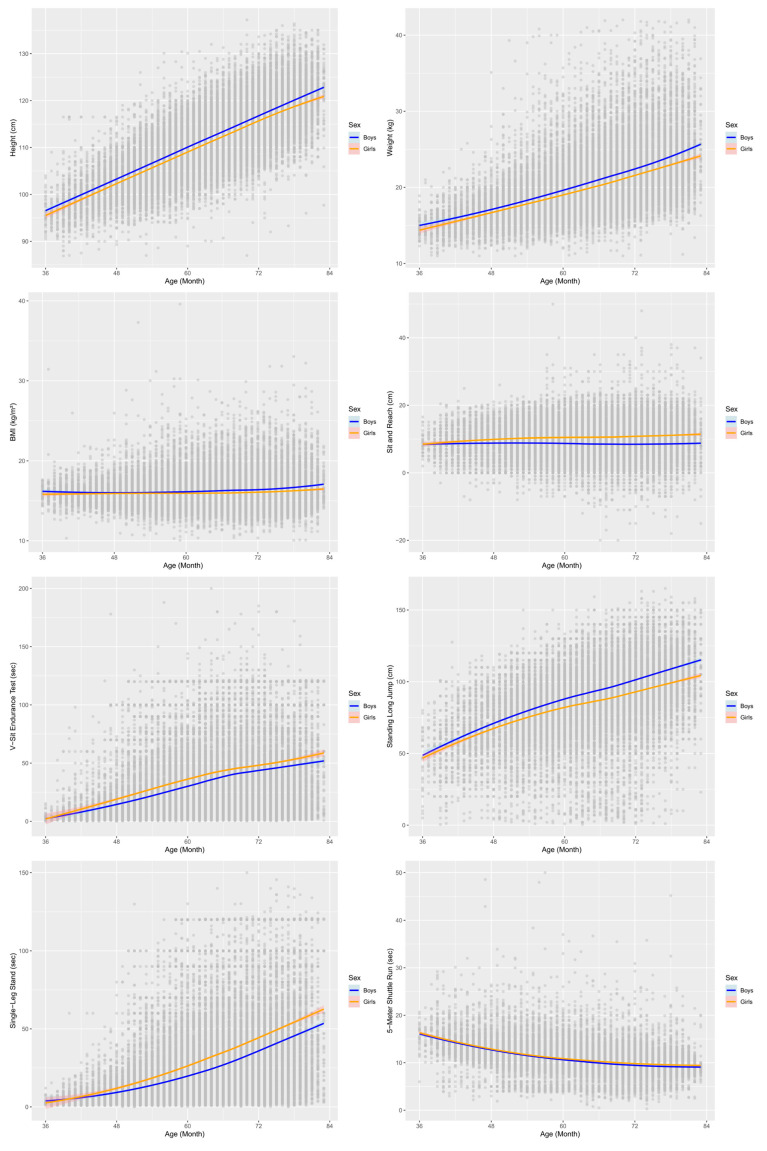
Graphs of anthropometric measures and physical fitness by age and sex (non-linear regression and 95% confidence intervals).

The patterns of physical growth and motor skill development by sex and age for Korean children aged 3–6 years are shown in Figure 2. Boys were taller and heavier than girls of the same age, and BMI was higher in boys. For motor skills, girls outperformed boys in the SRT, VSE, and SLS, while boys excelled in the SLJ. The 5SR slightly favored girls, but the difference was not significant.

The age- and sex-specific means, standard deviations, and 95% confidence intervals for physical fitness measurements are presented in Table 2. For SRT, boys showed a slight decrease from 8.78 cm to 8.5 cm with increasing age, whereas girls exhibited an increase from 9.5 cm to 10.9 cm. VSE demonstrated a substantial improvement in both boys and girls, increasing from approximately 8–10 s to 45–50 s. SLJ improved from approximately 60 cm to 100 cm, with boys consistently outperforming girls across all age groups. For SLS, boys improved from 6.2 s to 41.9 s, while girls increased from 7.2 s to 50.5 s, with girls outperforming boys at all ages. The 5SR showed a decreasing trend in both sexes, improving from approximately 14 s to below 9 s as age increased. Girls generally outperformed boys in flexibility, endurance, and balance, consistent with prior European studies. Boys exhibited greater explosive strength, particularly in the SLJ.

Compared to a previous large-scale study conducted in Asia, the SLJ results in this study showed that children jumped approximately 10 cm farther across all age groups [11]. In the SLS, performance was 1–5 s higher than in the Asian study and 2–20 s higher than in European studies [8,11]. However, the SRT results were largely similar to or lower than those reported in previous research. Due to differences in assessment methodologies, direct comparisons for other fitness tests were challenging.

**Figure 3:**
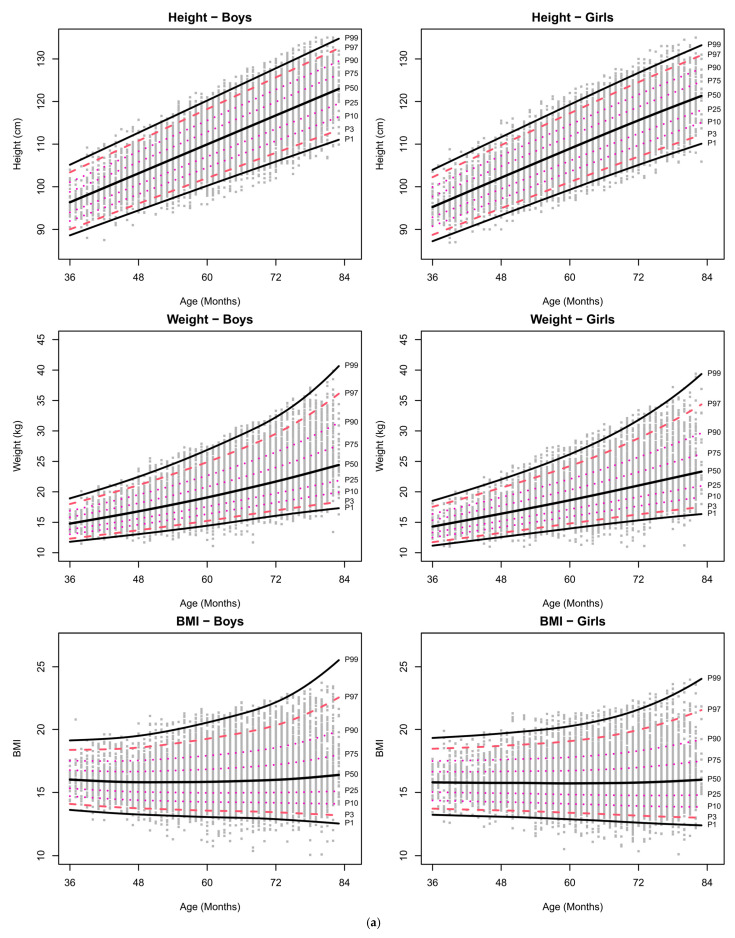

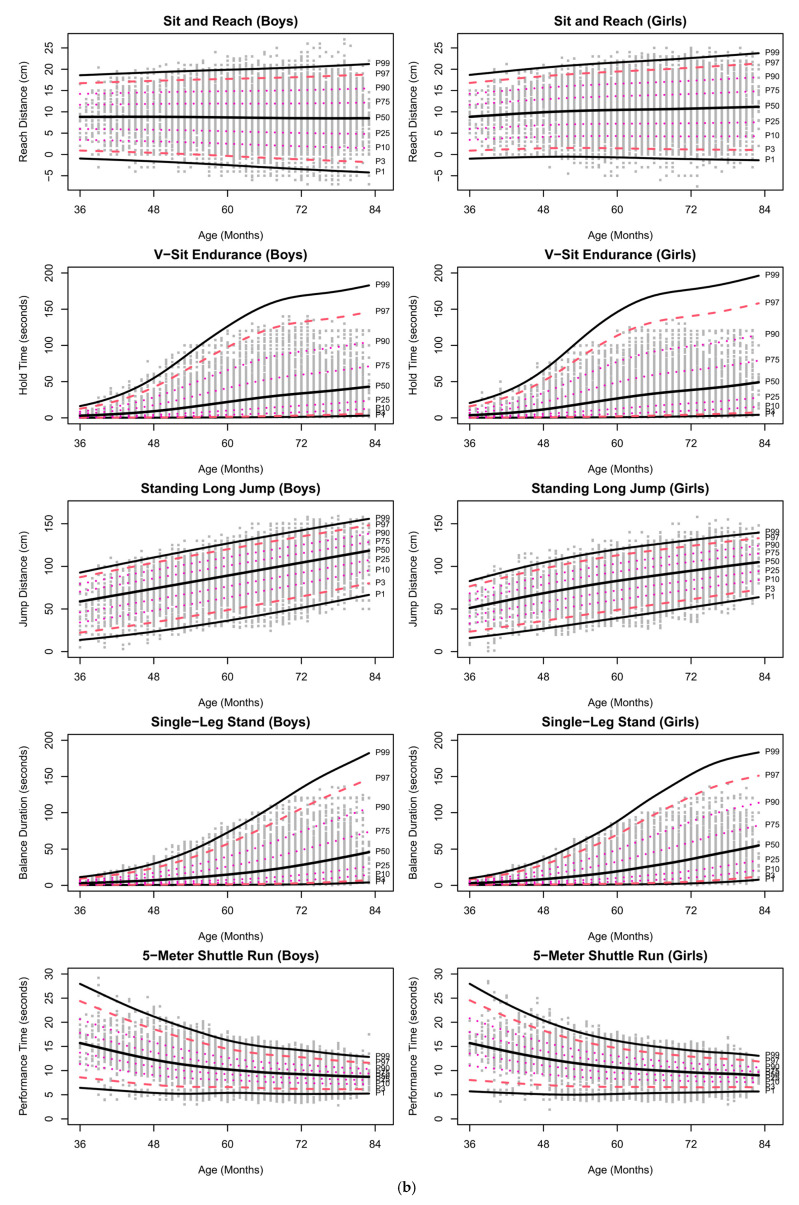
Age-related changes in anthropometric measures and physical fitness were modeled using GAMLSS. P1, P3, P10, P25, P50, P75, P90, P97, and P99 represent the 1st, 3rd, 10th, 25th, 50th, 75th, 90th, 97th, and 99th percentiles, respectively. (**a**) Percentile curves of anthropometric measures for Korean preschool children. (**b**) Percentile curves of physical fitness tests for Korean preschool children.

Age- and sex-specific percentiles, estimated using the GAMLSS method, are presented in Table 3 and Figure 3a,b. Table 3 provides the estimated sex-specific percentile values (1, 3, 10, 25, 50, 75, 90, 97, and 99) at key ages (36, 48, 60, 72, and 84 months). Figure 3a illustrates the percentile curves for anthropometric measurements, and Figure 3b displays the percentile curves for physical fitness measurements.

Overall physical fitness improved with age, but the SRT exhibited little change over time. The VSE and SLS showed greater variability with age, suggesting that differences in motor skills could widen over time. In contrast, the 5SR displayed decreasing variability with age, indicating a more uniform development pattern. Greater variability in endurance and balance with age suggests widening skill disparities. These findings are illustrated in Figure 3b and can also be observed in the changes in standard deviation values presented in Table 2.

**Figure 4:**
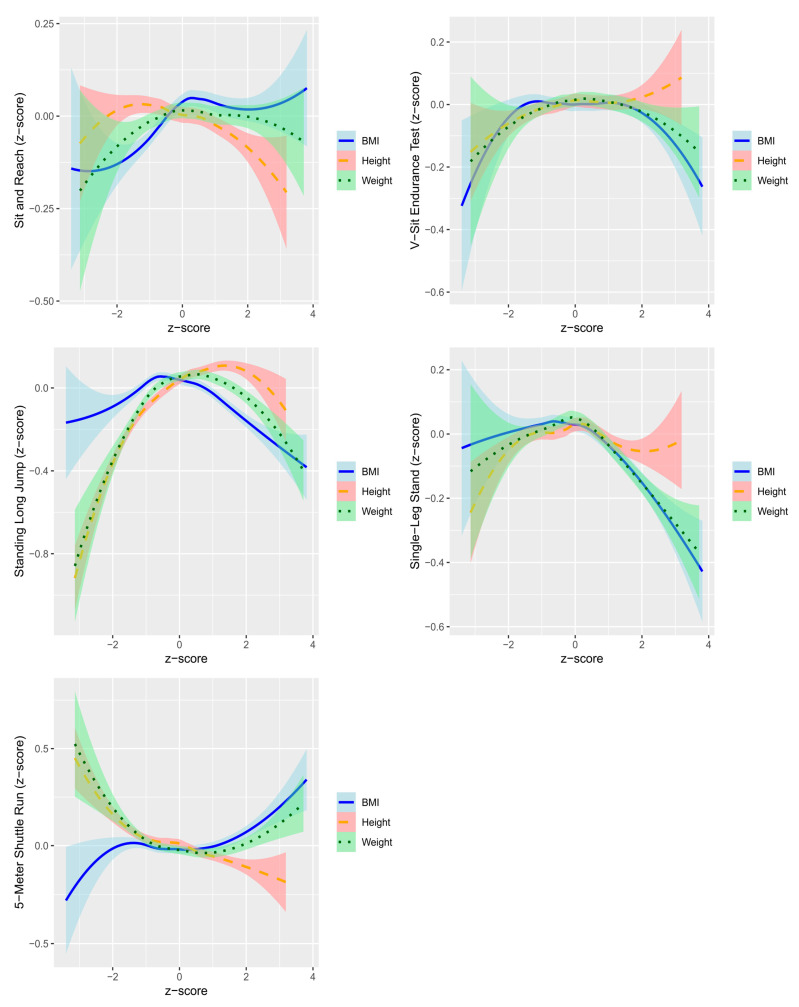
Non-linear relationships between fitness metrics and anthropometric measures using LOESS regression (95% confidence intervals).

The relationship between physical growth and motor skills is shown in Figure 4. Overall, the age-specific z-scores for physical fitness were highest when anthropometric values were close to the age-specific mean (z-score = 0). However, specific trends were observed: children with higher BMI performed better in the SRT, taller children performed better in the VSE, and lower BMI or taller stature was associated with better performance in the 5SR.

## 4. Discussion

### 4.1. International Context and Study Implications

Studies providing reference standards for physical fitness in preschool-aged children are limited [8,9,10]. However, previous research, such as a study conducted in China and Spain assessing similar fitness metrics, has reported findings that align with this study [8,11]. This may be attributed to the fact that the participants in this study were exclusively children attending daycare centers who are likely to have higher levels of physical activity and social engagement compared to the general preschool population.

This study analyzed data from approximately 40,000 children in Seoul, South Korea, representing one of the largest-scale investigations of this population. These fitness assessments provide valuable insights into the age- and sex-specific distribution of physical fitness during growth. The percentile curves presented in this study serve as critical benchmarks for assessing and monitoring the physical health of preschool children. These standards contribute to developing systematic health promotion strategies. Furthermore, the outcomes of each test play a crucial role in monitoring children’s overall developmental status, identifying areas requiring intervention, and providing appropriate support. These benchmarks enable educators and healthcare providers to identify children needing targeted motor interventions and to tailor physical education programs. For instance, children falling below the third percentile may require further evaluation of their activity levels and could benefit from additional exercise programs designed to enhance their physical fitness.

However, due to limitations in participant selection and the modeling approach, these findings should be interpreted with caution. Further research is needed to refine the models and improve the applicability of these reference standards.

### 4.2. Differences Between Boys and Girls

This study analyzed sex differences in physical characteristics and motor skills in preschool-aged children. The results showed that boys of the same age group were taller, heavier, and had higher BMI levels than girls. These findings align with the tendency of boys to develop muscle mass and physical stature more rapidly during this growth stage [27].

In motor skills, distinct differences were observed. Girls outperformed boys in the SRT, VSE, and SLS. Girls outperformed boys in the SRT by approximately 1–2 cm across all age groups, while the performance gap in the VSE and SLS widened with increasing age. Girls’ superior flexibility and balance align with European preschool studies, likely reflecting earlier neuromuscular development and greater engagement in fine motor activities [28,29]. Growing girls often exhibit better joint mobility and neuromuscular coordination, contributing to stronger motor skill performance [9].

Conversely, boys outperformed girls in the SLJ, which evaluates explosive lower-body strength and muscle power. This advantage may be attributed to their greater muscle mass and a higher capacity to develop explosive force, as well as more vigorous play behaviors [28,30,31]. Additionally, gendered play patterns and differing parental expectations in Korea may further contribute to these performance differences.

For the 5SR, girls showed slightly better results, though the differences between sexes were minimal. This may be because agility and cardiorespiratory endurance develop more uniformly with age, regardless of sex. Additionally, this test evaluates multidimensional motor skills, which could explain the negligible differences between boys and girls [13,30,31,32,33].

### 4.3. Age-Related Changes

The results of this study showed that overall physical fitness tends to improve with age. This can be attributed to the development of the musculoskeletal system and the improvement of neuromuscular coordination in children [34].

However, the SRT showed minimal changes with increasing age. This suggests that flexibility may not change significantly during the growth phase and may exhibit less variation than other physical abilities [29]. The minimal age-related gains in flexibility highlight the need for targeted stretching interventions, as maintaining or improving flexibility may require repetitive and systematic training [35].

The VSE and SLS exhibited increasing variability with age. This indicates that while some children show remarkable muscular endurance and balance development, others do not, highlighting individual differences [34]. These findings suggest that environmental, genetic, and activity-level differences become more pronounced in later preschool years [24,36]. In contrast, the 5SR tended to show decreased variability with age. This suggests that cardiorespiratory endurance and agility tend to develop more uniformly as children grow [37]. Agility-based tests like the 5SR may naturally improve through consistent physical activity and play, which could account for the reduced variation across ages [38].

In conclusion, this study demonstrates that while physical fitness improves with age, specific abilities may be less influenced by age or exhibit varying degrees of variability. These findings underline the complexity of physical development and the importance of targeted interventions to support balanced growth in children.

### 4.4. Relationship Between Anthropometric Measures and Physical Fitness

The results from this study, conducted on preschool-aged children, indicate that overall physical performance tends to be higher when height, weight, and BMI are closer to the average (z-score = 0). This finding highlights the importance of balanced growth in motor skill development.

However, certain physical attributes demonstrated specific and distinct relationships with particular motor skill performances.

For the SRT, children with higher BMI exhibited better performance in flexibility. This may be attributed to their increased body mass enabling a greater range of motion in the hamstrings and lower back. However, while a higher BMI may aid reach due to increased limb mass, excessive adiposity could potentially hinder overall movement and broader physical performance [4].

In the case of the VSE, taller children showed superior performance in core endurance. This finding suggests that greater height may offer a biomechanical advantage, aiding in balance and the ability to sustain isometric contractions during the test [39].

For the 5SR, children with lower BMI and greater height achieved better performance. Lower BMI likely reduces the metabolic demand during high-intensity activities, while greater height may contribute to longer strides and improved agility [4,13,40].

These findings highlight the nuanced and specific relationships between anthropometric factors and motor skills in preschool-aged children. They underscore the importance of understanding how physical attributes interact to influence motor performance at an early developmental stage [13].

### 4.5. Limitations

This study was conducted retrospectively using the provided data, which limited the available information for analysis. As a result, we were unable to account for factors that could potentially influence the participants’ physical fitness levels, such as activity levels, socioeconomic background, and COVID-19 lockdowns. Additionally, since the study population consisted solely of children attending daycare centers, it is possible that more active children were over-represented, leading to higher physical fitness scores compared to the general preschool population.

The uneven age distribution of the study participants posed challenges in optimizing the GAMLSS model, and in the case of the VSE, some degree of overfitting was suspected. Additionally, since the study was conducted exclusively on children in Seoul, the generalizability of the model’s results may be limited when applied to broader populations. However, compared to previous studies, this research was conducted on a large scale, and efforts were made to improve the results by removing outliers and comparing various models. Therefore, despite these limitations, the study findings may still provide valuable insights and have potential applications in relevant contexts.

In conclusion, this study provides comprehensive age- and sex-specific physical fitness benchmarks for Korean preschoolers, offering a vital tool for monitoring development and guiding early interventions. Findings emphasize the need for tailored physical activity programs to address sex differences and individual variability in motor skill development.

## Figures and Tables

**Table 1:** Overview of physical fitness tests and components.

Fitness Test	Abbreviation	Fitness Component	Unit of Measurement
Sit and Reach Test	SRT	Flexibility	Reach Distance (cm)
V-Sit Endurance Test	VSE	Abdominal Endurance	Hold Time (s)
Standing Long Jump	SLJ	Jumping Power	Jump Distance (cm)
Single-Leg Stand	SLS	Balance	Balance Duration (s)
5-Meter Shuttle Run	5SR	Agility	Performance Time (s)

**Table 2:** Result of physical fitness tests according to sex and age.

Age (Years)	Sit and Reach Test (cm)		V-Sit Endurance Test (s)		Standing Long Jump (cm)		Single-Leg Stand (s)		5-Meter Shuttle Run	
	Boys	Girls	Boys	Girls	Boys	Girls	Boys	Girls	Boys	Girls
3	8.78 ± 4.45 (8.48–9.09)	9.55 ± 4.39 (9.24–9.86)	8.24 ± 9.52 (7.56–8.93)	9.81 ± 10.34 (9.05–10.58)	63.37 ± 20.50 (61.95–64.78)	60.76 ± 18.57 (59.44–62.09)	6.25 ± 5.02 (5.90–6.61)	7.19 ± 5.87 (6.76–7.62)	13.75 ± 3.37 (13.52–13.99)	14.02 ± 3.36 (13.78–14.26)
4	8.79 ± 4.61 (8.66–8.93)	10.27 ± 4.69 (10.13–10.41)	22.16 ± 22.45 (21.48–22.83)	27.69 ± 25.50 (26.91–28.46)	81.47 ± 18.62 (80.92–82.02)	76.86 ± 16.70 (76.36–77.37)	14.00 ± 12.91 (13.62–14.39)	17.58 ± 14.87 (17.12–18.03)	11.23 ± 2.51 (11.16–11.31)	11.42 ± 2.44 (11.35–11.50)
5	8.57 ± 4.99 (8.46–8.69)	10.58 ± 4.91 (10.46–10.69)	37.15 ± 30.68 (36.46–37.85)	42.22 ± 31.29 (41.50–42.95)	95.61 ± 19.12 (95.17–96.04)	87.94 ± 16.92 (87.55–88.33)	26.02 ± 22.49 (25.51–26.53)	33.10 ± 24.77 (32.53–33.68)	9.90 ± 1.83 (9.86–9.94)	10.26 ± 1.82 (10.22–10.30)
6	8.50 ± 5.25 (8.35–8.66)	10.90 ± 5.22 (10.74–11.06)	45.86 ± 31.62 (44.91–46.81)	50.86 ± 31.99 (49.85–51.87)	107.26 ± 18.58 (106.70–107.82)	97.43 ± 16.98 (96.89–97.96)	41.87 ± 29.74 (40.98–42.76)	50.46 ± 30.74 (49.49–51.42)	9.19 ± 1.59 (9.14–9.23)	9.58 ± 1.57 (9.53–9.63)

Values are presented as mean ± standard deviation (95% confidence interval).

**Table 3:** Age- and sex-specific physical fitness percentiles in preschool children.

Age (Months)	Boys									Age (Months)	Girls								
	P1	P3	P10	P25	P50	P75	P90	P97	P99		P1	P3	P10	P25	P50	P75	P90	P97	P99
**Sit and Reach Test (cm)**										**Sit and Reach Test (cm)**									
**36**	−0.9	0.9	3.4	6.0	8.8	11.7	14.2	16.7	18.6	**36**	−1.0	0.9	3.4	6.0	8.8	11.7	14.3	16.8	18.7
**48**	−1.6	0.4	3.1	5.8	8.8	11.9	14.6	17.3	19.3	**48**	−0.6	1.4	4.1	6.9	9.9	12.9	15.7	18.4	20.4
**60**	−2.5	−0.4	2.5	5.4	8.7	11.9	14.9	17.7	19.9	**60**	−0.7	1.4	4.3	7.2	10.4	13.7	16.6	19.5	21.6
**72**	−3.5	−1.2	1.9	5.0	8.5	12.0	15.1	18.2	20.5	**72**	−1.1	1.2	4.2	7.3	10.8	14.2	17.3	20.3	22.6
**84**	−4.3	−1.9	1.4	4.8	8.5	12.2	15.5	18.8	21.3	**84**	−1.4	1.0	4.3	7.6	11.2	14.9	18.2	21.4	23.9
**V-Sit Endurance Test (s)**											**V-Sit Endurance Test (s)**								
**36**	0.1	0.2	0.6	1.4	3.0	5.5	8.6	12.6	16.2	**36**	0.1	0.3	0.7	1.7	3.7	6.8	10.8	15.9	20.5
**48**	0.2	0.5	1.6	4.1	9.1	17.5	28.2	42.2	54.9	**48**	0.3	0.8	2.2	5.3	11.6	21.6	34.4	50.9	65.8
**60**	0.6	1.4	4.2	10.0	21.9	41.2	65.8	97.6	126.3	**60**	0.8	2.0	5.5	12.6	26.7	49.1	77.4	113.6	146.1
**72**	1.4	3.2	7.9	16.9	33.7	59.6	91.6	132.2	168.4	**72**	2.1	4.4	10.0	20.1	38.4	65.6	98.9	140.4	177.3
**84**	3.3	6.2	12.9	24.4	44.0	72.3	106.1	147.9	184.6	**84**	4.5	8.0	16.0	28.8	50.3	80.6	116.3	160.1	198.4
**Standing Long Jump (cm)**											**Standing Long Jump (cm)**								
**36**	13.7	22.3	34.2	46.0	58.7	70.3	79.3	87.3	92.7	**36**	15.9	23.3	32.8	41.8	51.1	60.1	68.3	76.5	82.7
**48**	23.5	34.4	48.2	60.8	73.9	85.8	95.4	104.2	110.4	**48**	26.9	36.0	47.2	57.7	68.4	78.7	88.1	97.4	104.3
**60**	36.2	48.7	63.4	76.4	89.1	100.7	110.7	120.1	126.8	**60**	39.3	48.9	60.8	71.7	83.0	93.7	103.4	112.9	120.0
**72**	51.3	64.6	79.7	92.4	104.3	115.3	125.3	135.0	142.2	**72**	51.9	61.3	72.9	83.7	94.8	105.3	114.8	124.0	130.9
**84**	68.0	81.6	96.5	108.7	119.6	129.7	139.4	149.4	157.0	**84**	64.8	73.8	84.9	95.2	105.8	115.9	124.9	133.7	140.2
**Single-Leg Stand (s)**											**Single-Leg Stand (s)**								
**36**	0.3	0.6	1.1	1.8	3.1	4.8	6.8	9.2	11.4	**36**	0.5	0.7	1.2	1.9	3.0	4.5	6.1	8.0	9.7
**48**	0.5	1.0	2.1	3.9	7.1	11.7	17.1	23.9	29.9	**48**	0.6	1.2	2.5	4.7	8.5	14.0	20.5	28.6	35.7
**60**	0.7	1.5	3.6	7.5	14.8	26.0	39.8	57.2	72.7	**60**	1.1	2.2	5.1	10.2	19.3	32.8	49.3	69.8	88.0
**72**	1.4	2.9	7.0	14.4	28.1	48.6	73.9	105.8	134.0	**72**	2.6	5.0	10.6	20.0	36.2	59.6	87.7	122.4	152.9
**84**	4.3	7.7	15.3	27.4	47.6	76.1	109.6	150.7	186.5	**84**	8.5	13.2	22.5	36.0	56.8	84.3	115.6	152.9	184.8
**5-Meter Shuttle Run (s)**											**5-Meter Shuttle Run (s)**								
**36**	6.4	8.6	11.3	13.7	15.7	17.8	20.6	24.4	28.0	**36**	5.7	8.0	11.0	13.5	15.7	17.9	20.8	24.5	27.9
**48**	5.4	7.0	9.1	10.8	12.3	13.8	15.8	18.6	21.2	**48**	5.1	7.0	9.3	11.1	12.5	14.0	15.9	18.3	20.4
**60**	5.4	6.6	8.0	9.2	10.2	11.3	12.6	14.5	16.3	**60**	5.2	6.6	8.3	9.6	10.6	11.6	13.0	14.6	16.1
**72**	5.2	6.2	7.4	8.4	9.3	10.1	11.2	12.8	14.2	**72**	5.4	6.5	7.8	8.8	9.6	10.4	11.5	12.9	14.2
**84**	5.2	6.1	7.2	8.0	8.7	9.3	10.3	11.5	12.7	**84**	5.7	6.5	7.5	8.3	8.9	9.6	10.5	11.8	12.9

The percentile graphs for Korean children by age and sex, calculated using GAMLSS. Percentile values (1, 3, 10, 25, 50, 75, 90, 97, 99) for five physical fitness test items are presented at 36, 48, 60, 72, and 84 months.

## Data Availability

The datasets generated and/or analyzed during the current study are not publicly available due to restrictions of the Institutional Review Board of Seoul St. Mary’s Hospital, The Catholic University of Seoul, Korea, to protect patients’ privacy, but are available from the corresponding author upon reasonable request.
